# Primary High Grade Serous Carcinoma of the Fallopian Tube: A Case Report

**DOI:** 10.31729/jnma.5674

**Published:** 2020-11-30

**Authors:** Ramesh Dhakhwa, Anshu Vaidya, Amrita Giri, Archana Shakya, Achala Vaidya

**Affiliations:** 1 Department of Pathology, Norvic International Hospital, Kathmandu, Nepal; 2 Department of Obstetrics and Gynaecology, Norvic International Hospital, Kathmandu, Nepal

**Keywords:** *adnexal mass*, *fallopian tube*, *serous carcinoma*

## Abstract

A 49-year-old, perimenopausal nulliparous woman with lower abdominal pain and abnormal uterine bleeding. Clinical and radiological findings suggested a right adnexal tumor. CA-125 level was moderately elevated. Total abdominal hysterectomy with bilateral salpingo-oophorectomy was done. Peroperative findings revealed a soft to friable growth arising from right fallopian tube with no involvement of ovaries. Histopathologic examination confirmed it to be a high grade serous carcinoma, FIGO stage IA. The histomorphology resembled high grade serous carcinoma of ovary, however ovaries on both sides appeared unremarkable. Surgery was uneventful and the patient was discharged after seven days of hospital stay. She did not receive postoperative chemotherapy or radiotherapy and is under follow-up. The case is reported for its occurrence in an uncommon anatomic site and preoperative dilemma with relevant review of literature.

## INTRODUCTION

Primary fallopian tube carcinoma (PFTC) is extremely rare with an estimated incidence of 0.41/100000 population. High grade serous carcinoma is the commonest histopathologic type and has close resemblance to primary high grade serous ovarian carcinoma. Recent understanding of serous carcinogenesis suggest that almost all high grade serous carcinoma arise from the tubal epithelium irrespective of primary site and involves mutations in TP53. They are usually rapidly growing, highly aggressive neoplasms that are often diagnosed at an advance stage.^1^ Clinical presentation is however non-specific and may include lower abdominal pain, pelvic pain, serosanguinous vaginal discharge etc. Preoperative diagnosis is difficult and most of the cases are diagnosed per-operatively or following histopathologic examination.^2^ We present a case of high grade serous carcinoma in a 49 year old nulliparous woman.

## CASE REPORT

**Figure 1:**
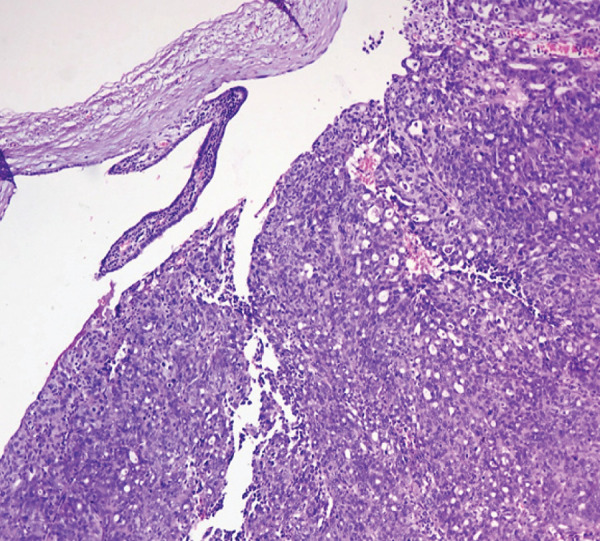
High grade serous carcinoma of fallopian tube (H and E; 100x magnification).

A 49 year old female presented with a history of lower abdominal pain and abnormal uterine bleeding. Physical examination did not reveal any significant findings. USG of abdomen suggested a right adnexal mass. Tumor marker (Ca 125) was moderately raised. Total abdominal hysterectomy with bilateral salpingo-oophorectomy was done and the specimen was submitted for histopathologic examination. Gross examination revealed an 8x7x5 cm mass arising from right fallopian tube. The mass was soft to friable. No tumor deposits were identified grossly on the ovarian or uterine surface. Histomorphologic examination revealed high grade serous carcinoma involving the right fallopian tube, FIGO stage IA (Figure 1).

Uterus and ovaries, left fallopian tube were unremarkable. Peritoneal fluid was negative for malignancy. In our case, USG findings revealed a right adnexal mass with no suspicion of fallopian tubal origin. It was only during per-operative examination, fallopian tubal origin of the tumor was identified which was confirmed by histopathology. The patient was discharged seven days after surgery and referred to oncologist for further management. She is however under gynaecologic follow up.

## DISCUSSION

Primary fallopian tube carcinoma is uncommon accounting for less than 1% of primary female genital tract malignancy. It typically presents in sixth to seventh decade of life and the patients are rarely 35 years of age. About 30% of the patients are nulliparous. The classic signs and symptoms of tubal carcinoma include vaginal bleeding, clear of serosanguinous vaginal discharge, pelvic pain and a pelvic mass. The diagnosis is rarely made before operation but occasionally positive cytology associated with negative endometrial curettage will lead to suspicion fortubal neoplasm. Tubal carcinoma may occur as a part of multifocal upper genital tract malignancy.^3–5^ PFTC resembles epithelial ovarian carcinoma (EOC) both clinically and histopathologically. PFTC is often diagnosed at an earlier stage than EOC due to abdominal pain from tubal distension and early symptoms.^6^

The rate of preoperative diagnosis is in the range of 0%-10%, and up to 50% are missed intraoperatively.^7,8^ PFTC should however be included in the differential diagnosis if the patient has clinical symptoms such as vaginal discharge or abnormal genital bleeding or spotting with negative diagnostic curettage. Pap smear positivity occurs in 10%-36% of cases.^9,10^ In our case, the patient was nulliparous and had history of abdominal pain and vaginal bleeding. Radiology revealed a right adnexal mass. Ca 125 was moderately raised. A suspicion of primary fallopian tubal origin was made only during per-operative evaluation and a final diagnosis of High grade serous carcinoma was made on histopathologic examination. Histopathology remains the gold standard in establishing the diagnosis of Primary fallopian tubal carcinoma and categorizing into its histologic subtypes. Recent understanding of the molecular nature of these tumors show TP53 mutation.^1^

The treatment of choice for PFTC is surgery and is similar to that for ovarian carcinoma. Postoperative platinum-based combination adjuvant chemotherapy is the most commonly used therapy for these patients, similar to EOC patients. The role of postoperative radiotherapy is even less clear. The stage of disease at the time of diagnosis is the most important factor affecting the prognosis. The other prognostic factors include the residual volume of the tumour after cytoreduction, the presence of ascites and the histologic grade of the tumor.^11,12^ The TNM/FIGO stage in our case was T1a/IA; suggesting a better prognosis.
